# Immune-Related Adverse Events: A Case-Based Approach

**DOI:** 10.3389/fonc.2019.00530

**Published:** 2019-06-20

**Authors:** Caoilfhionn Connolly, Kalindi Bambhania, Jarushka Naidoo

**Affiliations:** ^1^Department of Internal Medicine, Johns Hopkins Hospital, Baltimore, MD, United States; ^2^Department of Oncology, Sidney Kimmel Comprehensive Cancer Center at Johns Hopkins University, Baltimore, MD, United States; ^3^Department of Oncology, Bloomberg-Kimmel Institute for Cancer Immunotherapy, Baltimore, MD, United States

**Keywords:** immune checkpoint inhibitors, immune-related adverse events, immunotherapy, immune-related toxicities, management

## Abstract

Immunotherapy has heralded the advent of a new era in oncology. Immune checkpoint inhibitors (ICIs) enhance anti-tumor immunity, thereby reinvigorating a patient's immune system to fight cancer. While therapy with this class of agents has resulted in improved clinical outcomes for patients with multiple tumor types, a broad spectrum of immune-related adverse events (irAEs) may affect any organ system, with variable clinical presentations. Prompt recognition and management of irAEs are associated with improved irAE outcomes, and represents an important new clinical challenge for practicing oncologists. Herein, we provide a comprehensive case-based review of the most common and clinically-important irAEs, focussing on epidemiology, clinical manifestations, and management. We also examine future strategies that may provide meaningful insights into the prevention and management of irAEs.

## Introduction

Immune evasion is one of the hallmarks of cancer cells (1). Immune checkpoints are negative regulators of immune activation; exploiting the action of checkpoints such as cytotoxic T-lymphocyte antigen 4 (CTLA-4), programmed cell death (PD-1), and its ligand programmed cell death ligand 1 (PDL-1) allows cancer cells to evade immune-surveillance, thus enabling unchecked tumor growth. Immune checkpoint inhibitors (ICIs) negate this key mechanism of cancer progression, revitalizing the immune system to eradicate cancer cells. However, unleashing the effects of the immune system in this manner can result in a unique spectrum of toxicities known as immune-related adverse events (irAEs). ICIs have demonstrated unprecedented response rates in a wide array of cancer types, with seven checkpoint inhibitors currently approved by the FDA and in over 14 different cancer treatment indications (Table 1). As ICIs enter routine clinical practice, clinicians across all sub-specialties are increasingly faced with the diagnostic and therapeutic challenge of irAE identification and management.

**Table 1:** FDA-approved ICIs and their indications.

**Drug**	**Initial approval**	**Therapeutic target**	**Indication**
Atezolizumab	2016	PD-L1	Non-small-cell carcinoma Urothelial carcinoma
Avelumab	2017	PD-L1	Merkel cell carcinoma Urothelial cell carcinoma
Cemiplimab	2018	PD-L1	Cutaneous Squamous cell carcinoma
Durvalumab	2017	PD-L1	Urothelial carcinoma Non-small cell carcinoma
Ipilimumab	2011	CTLA-4	Melanoma
Nivolumab	2014	PD-1	Classical Hodgkin lymphoma Hepatocellular carcinoma Melanoma Microsatellite instability-high (MSI-H) or mismatch-repair deficient (dMMR) CRC Non-small cell lung cancer Renal cell carcinoma Small cell lung cancer Squamous cell carcinoma of head and neck Urothelial carcinoma
Pembrolizumab	2014	PD-1	Cervical cancer Classical Hodgkins lymphoma Gastric or Gastroesophageal junction adenocarcinoma Hepatocellular carcinoma Melanoma MSI-H or dMMR solid tumors Non-small cell lung carcinoma Squamous cell lung carcinoma Primary Mediastinal Large B-Cell Lymphoma Squamous cell carcinoma of head and neck Urothelial carcinoma
Ipilimumab and Nivolumab combination	2015	CTLA-4/PD-1	Melanoma MSI-H or dMMR colorectal carcinoma Renal cell carcinoma NSCLC

*PD-1, programmed cell death protein; PD-L1, programmed cell death ligand-1; CTLA-4, cytotoxic T lymphocyte-associated antigen 4*.

The incidence of irAEs has been reported to range from 15 to 90% in late phase clinical studies (2, 3). The risk, clinical manifestations, and severity of irAEs is variable across ICI regimen and cancer type. The frequency and severity of irAEs appears greatest amongst patients receiving ipilimumab/nivolumab combination therapy compared to monotherapy (4–6). Patients receiving CTLA-4 therapy more commonly present with colitis and hypophysitis, while patients receiving PD-1 therapy more commonly present with pneumonitis and thyroiditis (7–9).

**Figure 1:**
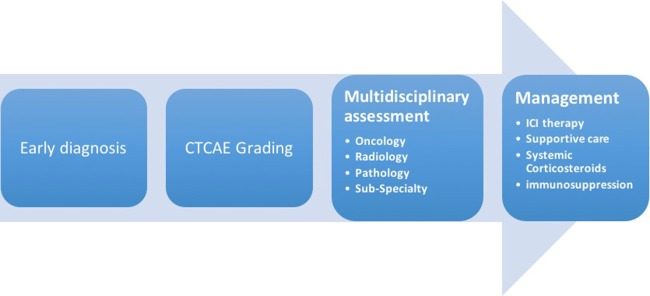
Early diagnosis and multidisciplinary management are integral to optimal clinical outcomes. It is crucial that physicians have a detailed knowledge of the wide range of clinical manifestations of irAEs, as well as familiarity with existing algorithms for their grading and management.

It is widely recognized that effective management of irAE is dependent on early recognition and prompt intervention (10). High clinical suspicion, timely evaluation and multi-disciplinary management provide the foundation for optimal clinical outcomes (Figure 1).

The severity of adverse events can be graded as per all adverse events, utilizing the Common Terminology Criteria for Adverse Events (CTCAE) (Table 2) (11). While this grading system has been used in oncology for many years, this is not specifically tailored toward irAEs, and should be supplemented by clinical judgement by those familiar with irAEs. In response to the clinical need to recognize and manage this class of toxicities, comprehensive guidelines to facilitate appropriate evaluation and management of irAEs were recently published by key oncology societies, including the American Society of Clinical Oncology (ASCO), the European Society for Medical Oncology (ESMO), the Society for Immunotherapy of Cancer Toxicity Management Working Group and the National Comprehensive Cancer Network (11–13). These guidelines are largely based on retrospective data and expert opinion. While they detail commonly encountered irAEs, rarer toxicities are not universally featured, and there is a paucity of guidance regarding severe or refractory irAEs, or those in which little has been published. Importantly, a recent meta-analysis demonstrated that fulminant irAEs resulting in patient death have been reported at a rate of 0.3–1.3% (14). Fulminant presentations are particularly challenging, and in the absence of prospective clinical data, management remains variable. While management varies according to severity of encountered irAE, ICI therapy can be continued for most grade 1 toxicities, with suspension of ICI for grade 2 or greater toxicities. Resumption of therapy following resolution of symptoms is a frequent clinical scenario that is currently lacking data. Retreatment can be considered for grade 2 or 3 toxicities when symptoms revert to grade 1 or less, however, permanent discontinuation of therapy is recommended with grade 4 irAEs (10, 12, 13). In a recent study of patients with NSCLC, retreatment with anti-PD-L1 therapy resulted in recurrence of irAEs in 52% of patients (15). The safety and benefit of retreatment is unknown and the decision to retreat should be considered on a case-by case basis.

**Table 2:** Common terminology criteria for adverse events grading.

**CTCAE grade**	**Level of care**	**Management**	**ICI therapy**
1: Asymptomatic or Mild	Ambulatory	Observation	Continue ICI therapy with close monitoring
2: Moderate	Ambulatory	Systemic corticosteroids (0.5–1 mg/kg/day of prednisone or equivalent)	Temporarily hold; resume when grade 1 or less
3: Severe but not immediately life-threatening	Inpatient	High dose systemic corticosteroids (1–2 mg/kg/d prednisone or methylprednisolone); consider additional therapies if no response with 48–72 h	Temporarily hold; resume when grade 1 or less in discussion with patient
4: Life-threatening	Inpatient +/– intensive care unit	High-dose corticosteroids (1–2 mg/kg/d prednisone or methylprednisolone); consider additional therapies if no response with 48–72 h	Permanent discontinuation with the exception of endocrinopathies managed by hormone replacement

This review provides a practical guide to what is known about irAEs and how to optimize evaluation and management of irAE utilizing a case-based approach. Within common or rare but clinically-important cases, we highlight the current state-of the art in terms of diagnosis and management, as well as emerging challenges. Finally, we discuss new therapies and novel approaches that may alleviate the future burden of irAE.

## Organ-Specific irAEs

**Figure 2:**
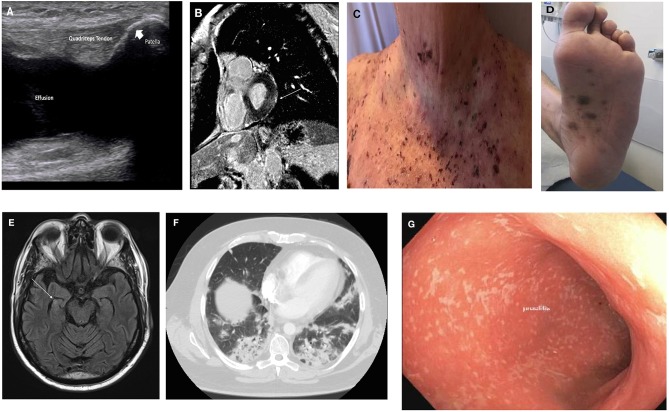
Clinical manifestations of irAE. **(A)** Inflammatory arthritis: US image of knee effusion; **(B)** myopericarditis: cardiac MRI with late gadolinium enhancement overlying basal left ventricular lateral wall (arrow); **(C)** bullous pemphigoid; **(D)** lichenoid dermatitis; **(E)** encephalitis: brain MRI with hyperenhancement of right hippocampus (arrow); **(F)** pneumonitis: chest CT with bilateral lower lobe infiltrates; **(G)** colitis: endoscopic findings of pan-colitis.

Given the wide array of clinical manifestations of irAEs, we summarize the diagnosis, management and emerging data in the most common or clinically-challenging irAEs that clinicians may encounter by organ system. Figure 2 illustrates some of the irAE manifestations encountered at the Johns Hopkins Hospital.

## Rheumatic

### Case 1: Inflammatory Arthritis

*Clinical Presentation: A 58-year old woman is treated with combination Ipilimumab/Nivolumab therapy for stage IV non-small-cell lung carcinoma (NSCLC), and presents with a 3-week history of a left, swollen, and painful left knee*.

Arthralgia and inflammatory arthritis (IA) are the most commonly encountered rheumatic irAEs (16, 17). ICI-induced IA can have a variable timing, with a median onset 5 months (range 1–24 months) after ICI initiation (18). The clinical presentations of IA are variable with oligoarthritis, polyarthritis, and reactive arthritis-like manifestations being described (19). Cappelli et al. reported that patients who received combination ICIs were more likely to present with large joint involvement and to already have another irAE, while patients treated with ICI monotherapy were more likely to have initial small joint involvement and to have IA as their only irAE (16). Other rheumatic irAEs include polymyalgia-like syndrome, vasculitis, sicca syndrome, and inflammatory myopathies (20). The data regarding the incidence of rheumatic complications is highly variable, with rates of arthralgia ranging from 1 to 43%, with reports of other manifestations ranging from 0.7 to 5.1% (21, 22). This is potentially related to the variability in potential coding of these events in clinical trials, using CTCAE criteria.

#### Diagnostic Evaluation

Patients should undergo a full musculoskeletal evaluation. Laboratory studies including ESR (Erythrocyte sediment rate), CRP (C-reactive protein), RF (rheumatoid factor), ACPA (anti-citrullinated peptide/protein antibodies), ANA (anti-nuclear antibody), and HLA-B27 (Human Leukocyte Antigen B-27) should be sent, to help differentiate between phenotypes of IA that may have treatment implications. The majority of patients are seronegative, but a seropositive subgroup has been described (16). Imaging including joint ultrasound or MRI should be completed to assess for effusion, erosive disease and tenosynovitis. In the above patient, diagnostic evaluation was notable for left knee effusion (see Figure 2A) with unremarkable synovial fluid analysis, elevated inflammatory markers, and seronegative disease.

#### Management

Early recognition of IA is critical to avoid erosive joint damage. ICI can be continued for grade 1 toxicities and treated with simple analgesia consisting of acetaminophen and non-steroidal anti-inflammatory drugs (NSAIDs). However, in this patient with grade 2 IA, ICI was temporarily held while she received treatment with acetaminophen, NSAIDs and oral corticosteroids of prednisone 20 mg/day for 4 weeks. Given large joint involvement, she also received an intra-articular corticosteroid injection. Corticosteroid dose can be adjusted pending clinical response with taper over 4–6 weeks in the instance of clinical improvement, or, in presence of progressive IA, treatment can be escalated with addition of other immunomodulatory medications such synthetic or biologic disease-modifying anti rheumatic drugs (DMARDs). This patient demonstrated improvement and prednisone was tapered over 4 weeks. Ipilimumab/Nivolumab therapy was resumed when IA symptom control was reached, and the patient was taking a prednisone dose of 10 mg daily. A notable feature of rheumatic irAEs, in particular ICI-induced-IA, is their predilection for persistence despite cessation of ICI therapy which may require long-term immunomodulatory therapy for months to years after diagnosis (23). Due to likely prolonged treatment requirements, physicians should consider starting immunomodulatory medications earlier than one would with other irAEs. DMARDs should be considered in all patients with grade 3 or 4 disease, as well as grade 2 patients that display progression of disease with corticosteroid therapy. In steroid-refractory cases, a common approach is to start with Methotrexate, with escalation to biologic agents such as infliximab in the absence of adequate clinical response.

### Case 2: Temporal Arteritis

*Clinical Presentation: A 76-year old man with advanced urothelial presents with temporal headache and jaw claudication 10 days after cycle two of durvalumab*.

Both polymyalgia-like syndrome and giant cell arteritis (GCA) have been reported following treatment with ICI. A recent analysis of WHO's VigiBase found that patients who received ICI had a reporting odds ratio of GCA 13 times greater than patients not treated with ICI (24). This study also reported that the median time of onset from last dose of ICI was 55 days (range: 21–98) with a greater predilection for elderly patients, Caucasian patients, and those with melanoma (24). ICI-induced GCA symptoms mirror those of traditional GCA, including temporal headache, jaw claudication, monocular vision loss, unexplained fever, and fatigue.

#### Diagnostic Evaluation

Early diagnosis is vital to prevent devastating ocular and cerebrovascular complications of GCA. Visual impairment has been reported in 28% of patients with ICI-induced GCA (24). The diagnosis of GCA should not be made based upon symptoms alone and investigations including complete blood count (CBC), ESR, and CRP. Temporal artery biopsy is the gold standard diagnostic test and provides definitive diagnosis, but should not delay treatment. In this patient, physical examination was notable for temporal artery tenderness with intact vision. Initial investigations were notable for markedly elevated ESR and CRP.

#### Management

Given intact vision, the patient was commenced on prednisone 60 mg/day to complete 2 weeks of therapy followed by a taper every 2 weeks. Durvalumab was held pending clinical response. Temporal artery biopsy confirmed the diagnosis. This management was instituted with the input of a rheumatology consult.

Patients with suspected ICI-induced GCA should be managed as per traditional rheumatic GCA with the addition that ICI therapy should be held pending clinical improvement in GCA. In patients without visual loss at diagnosis, treatment should comprise prednisone 1 mg/kg/day (maximum dose of 60 mg daily). Patients with threatened or established visual loss at diagnosis should be commenced on intravenous pulse corticosteroids (1 g methylprednisolone daily) for 3 days followed by high dose oral therapy (25). Oral corticosteroids should be maintained for 2–4 weeks followed by a two-weekly interval taper. Typically, prednisone will be tapered by 10 mg every 2 weeks until a dose of 40 mg/day is reached, at which point the dose will be reduced by 5 mg decrements every 2 weeks until a dose of 20 mg/day is reached. At this point, the rate of the corticosteroid taper is slowed.

In the case of relapsed GCA symptoms despite high-dose corticosteroids, abatacept has demonstrated efficacy (26), while methotrexate and tocilizumab are alternate therapeutic options. Resumption of ICI therapy can be considered once prednisone dose is <10 mg/day, ideally in consultation with rheumatology.

## Cardiovascular

### Case 1: Myocarditis

*Clinical presentation: A 65-year old man with advanced renal cell carcinoma presents with chest pain and dyspnea following 1 cycle combination Ipilimumab/Nivolumab therapy*.

Myocarditis is the most commonly documented cardiac irAE (27). Cardiovascular complications of ICIs are less well-recognized, but these complications can be potentially fatal (24, 28). The absolute incidence of cardiac irAE is reported at <1%, however the true incidence is likely higher given prior under-recognition of cardiac toxicity (26). Recently, Salem et al. reported that the odds of myocarditis in patients receiving ICIs was 11 times greater than patients who did not receive ICI (24), with a median time of onset 30 days after initial exposure to therapy. Wang et al. recently found that myocarditis has the highest fatality rate of any irAE (14). Manifestations of myocarditis are variable, with a clinical spectrum ranging from fatigue, chest pain, acute heart failure to cardiogenic shock, arrhythmias, and sudden death (29, 30). Pericarditis, conduction disease and ventricular arrhythmias are other reported cardiac irAEs, but acute myocardial ischemia, new onset systolic dysfunction and Takotsubo syndrome can also occur (31).

#### Diagnostic Evaluation

Myocarditis is characterized by elevated cardiac enzymes (troponin, pro-BNP), with/without the onset of left ventricular dysfunction and evidence of myocardial inflammation on cardiac MRI or PET/CT; all of these parameters should be investigated. In cases of uncertainty, endomyocardial biopsy can be useful although non-invasive investigations are preferred. Initial evaluation of this patient was notable for marked hypervolemia and pulmonary edema. Diagnostic workup was notable for non-specific ST-segment changes on ECG, marked elevation of cardiac markers and new reduced ejection fraction on transthoracic echocardiogram. Cardiac MRI was notable for late gadolinium enhancement overlying the basal left ventricular lateral wall (see Figure 2B).

#### Management

The patient was transferred to the Cardiac Care Unit under the care of the cardiology team. ICI was permanently discontinued. The patient was monitored on continuous telemetry and commenced treatment with daily intravenous 1 g methylprednisolone and diuretic therapy. His hospital course was complicated by complete heart block that was managed with transvenous pacing, however, progressive clinical deterioration followed, resulting in cardiac arrest from which he could not be resuscitated.

All grades of cardiac toxicity warrant evaluation given the risk of cardiac compromise. Effective management requires close monitoring with a multimodal therapeutic plan consisting of ICI cessation, high-dose corticosteroids (1–2 mg/kg of prednisone/day or equivalent) and early cardiology consultation with management of cardiac complications. Steroid-refractory cases may necessitate the addition of mycophenolate, infliximab, or anti-thymocyte globulin, and there are no specific data that demonstrate a superior approach of these three options. Conduction disease is emerging as a common and potentially serious cause of ICI-medicated sudden death, even in the absence of myocarditis (32). Electrophysiology consultation should be completed for consideration of insertion of cardiac device (pacemaker or defibrillator) if there is concern for ICI-induced conduction disease. In the instance of cardiac device insertion, the decision to proceed with ICI therapy should be made in conjunction with the patient, cardiology, and oncology. A surveillance strategy has recently been proposed that suggests a baseline cardiac assessment for all patients including baseline cardiac risk factor assessment, electrocardiogram, cardiac troponin, and pro-brain natriuretic peptide (pBNP) in addition to a non-invasive surveillance protocol for patients with cardiac risk factors defined as pre-existing coronary artery disease, hypertension, diabetes mellitus, obesity, smoking history, or positive family that should be completed within the initial 12 weeks of therapy (32). Given the mortality risk of these complications this approach may be appropriate, but should be evaluated in prospective studies.

## Dermatologic

### Case 1: Maculopapular Rash

*Clinical presentation: A 61-year old woman with Merkel cell carcinoma presents with a pruritic rash after cycle two of Avelumab therapy. She denies mouth pain, eye pain, fever, or constitutional symptoms*.

Cutaneous toxicities are the most commonly encountered irAE, and has been reported in 30–50% of patients receiving ICI therapy (33), with 37–70% of patients receiving CTLA-4 and 17–37% of patients receiving PD1/PDL-1 therapy experiencing dermatologic toxicities, respectively (2, 3). Of these, <3% experience greater than grade 3 toxicity. Dermatologic irAEs are challenging as they have variable clinical presentation and timing of onset. Clinical manifestations range from pruritus, vitiligo, inflammatory rashes (maculopapular eruption, dermal hypersensitivity reactions, acneiform, exfoliative, and psoriasiform lesions), bullous dermatoses (bullous pemphigoid, bullous drug reaction) to severe cutaneous adverse reactions (Stevens Johnson Syndrome, Toxic Epidermal Necrolysis, Drug-induced hypersensitivity syndrome/Drug reaction with eosinophilia and systemic symptoms). Time to onset can vary between 2 weeks and several months from onset of therapy (34, 35).

#### Diagnostic Evaluation

In patients with cutaneous irAE, a thorough clinical history and physical examination should be obtained. Clinicians should perform a close evaluation of all skin surfaces, mucus membranes, and lymph nodes with a specific focus on the percentage body surface area that is involved and the presence or absence of blistering. A positive Nikolsky sign (induction of blistering via mechanical pressure) should prompt concern for severe cutaneous reaction (SJS/TEN), which will characteristically include mucosal and systemic involvement (fever, constitutional symptoms). On examination, this patient was noted to have tense blisters and erosions on her extremity flexures that involved 15% body surface area. Nikolsky sign was positive. There was no evidence of ocular of mucosal involvement. Hematological and biochemical investigations were normal. She was evaluated by a dermatologist and underwent lesional and perilesional biopsies that confirmed the diagnosis of bullous pemphigoid. Skin biopsy revealed linear immunoglobulin G (IgG) and linear C3 staining along the basement membrane zone, which is present in >90% of cases (36). If biopsy is not possible, serum can be sent for antibodies to BP180 and BP230 (ELISA testing) to confirm the diagnosis (36).

#### Management

The patient was treated with betamethasone topical therapy and oral prednisone 1 mg/kg, tapered over 4 weeks. The patient was monitored closely with serial photography for progression but displayed evidence of improvement. Avelumab was initially held, but resumed upon resolution of symptoms to less than grade 1 severity.

With the exception of bullous disease, most grade 1 and 2 cutaneous toxicities can be managed with topical therapies (emollients, corticosteroids) and continuation of immunotherapy. Escalation of dermatologic care includes holding immunotherapy, increasing the potency of topical corticosteroids and starting systemic corticosteroids. Grade 4 toxicities should be treated with intravenous methylprednisolone dosed at 1–2 mg/kg. Patients with >30% body surface area involvement should be managed in specialist burns unit. In steroid-refractory cases, IVIG or cyclosporine can be considered in conjunction with dermatology. Notably, cutaneous irAEs have been recognized as a barrier to ICI compliance (11). Interestingly, development of cutaneous toxicities may correlate with clinical response in patients with metastatic melanoma, with a greater survival benefit reported in melanoma patients who developed rash or vitiligo after pembrolizumab or nivolumab therapy, respectively (37, 38). Development of new vitiligo has also demonstrated a significant association with both progression-free survival and overall survival in a meta-analysis of 27 studies of melanoma patients treated with a wide variety of immunotherapeutic strategies (139 treatment arms comprising 11 general immune stimulation, 84 vaccine, 28 antibody-based, and 16 adoptive transfer)(39).

## Renal

### Case 1: Nephritis

*Clinical presentation: A 58-year old woman with stage IV PD-L1*+ *NSCLC was noted to have asymptomatic creatinine (Cr) elevation to 2.5 mg/dl (baseline 0.9 mg/dl) after 2 cycles of Pembrolizumab*.

Nephritis is the most common renal toxicity of anti-PD-1/PD-L1 therapy, and is more common in patients with NSCLC treated with the combination of chemotherapy and immunotherapy, which is now standard first-line therapy for patients with advanced NSCLC (40). Hyponatremia may also be encountered in these cases, however this occurs more commonly in the setting of hypophysitis (41). There is significant heterogeneity in the onset of kidney injury; with CTLA-4 nephrotoxicity occurring earlier (range: 2–3 months), compared to the later onset of injury with PD-1 related nephrotoxicity (range: 3–10 months) (42–44). Acute interstitial nephritis is the most prevalent pathologic lesion, with one report of thrombotic microangiography (41). While initial data suggest that ICI-mediated renal injury ranged from 1 to 2% in monotherapy and 4.5% in combination therapy (45), more recent studies have suggested a higher incidence ranging from 9.9 to 29% (46).

#### Diagnostic Evaluation

Patients with renal irAEs are frequently asymptomatic and therefore, routine monitoring of renal indices (serum creatinine, electrolytes) is necessary to ensure prompt detection. Symptomatic patients may present with nausea, vomiting, fatigue, altered mental status, reduced urinary output, peripheral edema, or dyspnea. All patients should undergo complete renal evaluation including urinalysis, serum creatinine, serum electrolytes, and consideration for renal ultrasound to evaluate for other potential etiologies. In this asymptomatic patient with Cr 2.5 mg/dl, urinalysis was notable for pyuria with mild peripheral eosinophilia on CBC. Investigations may reveal pyuria (68%), hematuria (16%), and/or proteinuria on urinalysis with eosinophilia (21%) on CBC (41, 42).

#### Management

Therapy should be temporarily withheld while evaluation for an underlying cause of nephrotoxicity is completed. Should no alternate cause be identified, patients should be presumed to have immune-mediated toxicity. Reflex kidney biopsy is not recommended until corticosteroid treatment has been attempted. Corticosteroids are the mainstay of treatment. Additional immunosuppression including mycophenolate can be considered in steroid-refractory cases. In this patient, Pembrolizumab was held temporarily. Nephrology were consulted and she was commenced on prednisone 1 mg/kg/day. At 2-week follow up Cr had improved to 1.8 mg/dl. Prednisone was tapered over 4 weeks with improvement in renal indices to baseline. Following discussion with the patient regarding risks and benefits, pembrolizumab was resumed.

## Neurology

### Case 1: Encephalitis

*Clinical presentation: A 62-year old female with stage IV renal cell carcinoma presents with headache and altered mental status 1 week following Ipilimumab/Nivolumab therapy*.

Neurological toxicities, while uncommon, are of special interest due to their potential severity. These complications encompass dysregulation of both central and peripheral nervous systems. Central manifestations include encephalitis, aseptic meningitis, transverse myelitis and posterior reversible encephalopathy syndrome (PRES). Encephalitis is estimated to occur in 0.1–0.2% of patients (47). Patients may present with headache, altered mental status, motor or sensory deficits, abnormal behaviors, personality change, speech disorders, or movement disorders. A meta-analysis of 9,208 patients who received ICI therapy reported that incidence of neurologic irAEs ranged from 3.8 to 6.1% with anti-CTLA4 and anti-PD-1 monotherapy, respectively, and up to 12.0% with combination therapy. High-grade events were reported at an incidence of <1% across all ICIs (47). The median time of onset to encephalitis is 6 weeks. Most neurological irAEs present initially with non-specific symptoms such as headache, dysgeusia and sensory impairment (47, 48).

#### Diagnostic Evaluation

A baseline complete blood count, liver, renal, and thyroid function should be sent as well as assessment of the pituitary axis. If there is concern for a vasculitic process, ESR, CRP, and ANCA should be sent. Lumbar puncture should be completed to evaluate for infection and leptomeningeal disease. Anti–N-methyl-D-aspartate receptor (anti-NMDAR) antibody should also be sent. Contrast-enhanced MRI brain scan and EEG monitoring can be completed to assesses for vascular insult, brain metastasis and subclinical seizure activity. In this patient, the diagnostic evaluation revealed an intact pituitary axis, with a subtle hyperintensity of the right hippocampus on MRI (see Figure 2E). CSF analysis was notable for a lymphocytic pleocytosis with negative infectious, paraneoplastic and cytopathology panel. EEG showed diffuse non-specific slowing.

#### Management

Prompt recognition and expeditious management carries the potential of complete neurological recovery (48, 49). In this patient, ICI was held and she was commenced on IV acyclovir that was discontinued on receipt of negative viral PCR panel. Neurology were consulted. She was treated with pulse corticosteroids (methylprednisolone 1 g IV daily for 5 days) as well as IVIG 2 g/kg over 5 days. Following 8 days of therapy with corticosteroids and IVIG, she demonstrated full neurological recovery. A recent case series of nine patients with ICI-induced neurological toxicity reported that 77.8% of patients showed marked symptomatic improvement following discontinuation of immunotherapy and management with corticosteroids (49). In steroid-refectory cases, additional therapeutic modalities such as IVIG, rituximab or plasmapheresis can be considered, since this toxicity is autoantibody-mediated.

### Case 2: Myasthenia Gravis

*Clinical Presentation: A 67-year old man with metastatic colorectal cancer presents with diplopia 1 week following the first cycle of Nivolumab therapy*.

Myasthenia gravis is estimated to occur in 0.1–0.2% of patients receiving immunotherapy (50, 51). Presentation is typically within 2–3 weeks of treatment initiation (52, 53) with symptoms of fluctuating motor weakness and fatigue that is often associated with ocular and bulbar dysfunction. Peripheral neurotoxicity can also manifest as peripheral neuropathy, autonomic neuropathy, Guillain-Barre syndrome, and necrotizing myositis.

#### Diagnostic Evaluation

Any concern for myasthenia gravis warrants rapid evaluation and intervention given the potential for respiratory compromise. In this patient, acetylcholine receptor antibodies were positive. Serial pulmonary function test with negative inspiratory force (NIF) and vital capacity did not reveal respiratory compromise. Electrophysiologic testing (Single fiber EMG) confirmed the diagnosis of myasthenia gravis. Other investigations including creatinine kinase, aldolase, ESR, and CRP should be sent to assess for concurrent myositis. High clinical suspicion for concurrent myositis and myocarditis is warranted given possibility of coexisting myasthenia gravis, myositis and myocarditis in a subset of patients as evidenced in 25% of cases of nivolumab-related myasthenia gravis (52).

#### Management

ICI-induced myasthenia gravis has been associated with a higher incidence of myasthenic crisis than idiopathic myasthenia gravis (52, 53). Thus, a high level of suspicion and rapid initiation of corticosteroids are mandatory to prevent clinical deterioration, which can result in respiratory failure and death. In this patient, nivolumab therapy was held. Neurology were consulted and he was commenced on pyridostigimine 30 mg four times per day as well as prednisone 1 mg/kg/day. With clinical improvement, prednisone was slowly weaned in 5 mg decrements every 2 weeks. Patients with grade 3 or 4 toxicity should be monitored in the intensive care setting given risk of respiratory compromise. Pyridostigimine can be titrated to achieve optimal relief of symptoms. Additional therapeutic modalities include IVIG or plasmapheresis that should be initiaited for grade 3 or 4 disease.

## Ocular

### Case 1: Uveitus

*Clinical Presentation: A 71-year old man with history of urothelial cancer noted onset of right eye pain 5 weeks following cycle 1 of Atezolizumab therapy*.

Ophthalmic irAEs affect <1% of ICI-treated patients and typically manifest as uveitis and/or dry eye (54, 55). Ocular irAE have a median onset of 2 months and are more commonly associated with other concurrent irAEs (55). Patients can present with eye pain, erythema, pain with eye movement, visual disturbance, diplopia, or photophobia. Less commonly encountered ocular manifestations include inflammatory orbitopathy, keratitis, choroidal neovascularization, serous retinal detachment, retinopathy, neuroretinitis, and ocular myasthenia gravis.

#### Diagnostic Evaluation

Ocular irAEs are commonly seen in associated with other systemic irAEs, therefore clinical suspicion for other manifestations should be high. This particular patient endorsed the presence of visual floaters, but denied pain with eye movement, change in color perception, visual change, or photophobia. On examination, the patient's right pupil was mildly constricted, reactive to light with erythema of the limbus. Left pupil was round and reactive. Color vision and visual acuity were intact. Red reflex was present bilaterally. There was no evidence of concurrent irAE.

#### Management

The patient was prescribed topical corticosteroid, 1% cyclopentolate (topical cyclopegic agent) and prednisone 60 mg daily. An urgent ophthalmology appointment was scheduled within a week. Atezolizumab was temporarily held until completion of corticosteroid taper over 2 months.

The majority of ocular irAE do not necessitate discontinuation of ICI and are managed with topical therapies. All patients should be referred to ophthalmology for slit-lamp and dilated fundus examination to assess for presence of leukocytes in the anterior chamber of the eye as well as to examine the extent of inflammation. In this case, ICI was temporarily held in the setting of grade 2 toxicity. However, ICI should be permanently discontinued with emergent ophthalmology assessment in higher grade irAEs. Additional therapeutic strategies include systemic and topical/intravitreal corticosteroids. Infliximab can be considered in steroid refractory cases (56).

## Pulmonary

### Case 1: Pneumonitis

*Clinical presentation: A 54-year-old man with advanced urothelial carcinoma presents following the fifth dose of Durvalumab with dyspnea*.

Checkpoint-inhibitor pneumonitis (CIP) is defined as the development of new infiltrates on chest imaging with dyspnea or other respiratory symptoms in the absence of infection, cardiac dysfunction or tumor progression. Presentations can be heterogeneous, ranging from asymptomatic radiographic findings, chest pain, cough, or dyspnea, to life-threatening respiratory compromise (57). The overall incidence of CIP ranges from 0 to 10%, with a median time to onset of ~3 months reported by Naidoo et al. (8). Patients receiving combination ICI therapy are at increased risk of CIP (10 vs. 3%, respectively; *p* < 0.001), with evidence to suggest that these patients experience symptoms earlier in the clinical course (8, 57). In a study of fatal ICI-associated toxic effects, anti–PD-1/PD-L1–related fatalities were often from pneumonitis, consisting of 35% of all fatalities (14). The data would suggest that higher grade CIP tend to occur within the first 100–200 days of therapy initiation (57). Emerging data from the Johns Hopkins Hospital group has shown that tumor histology may be a risk factor for CIP in NSCLC patients (58). Furthermore, multistate modeling has demonstrated that NSCLC patients who develop CIP may have a poorer survival (59).

#### Diagnostic Evaluation

The patient underwent thorough history and physical examination that was notable only for hypoxia with O_2_ saturation of 88% on room air. Physical examination in CIP can be very unrevealing and thus clinicians must be vigilant for early detection. The differential diagnosis should include respiratory infection, rare respiratory infections such as PCP or aspergillosis (especially if being treated with high-dose corticosteroids), tumor progression, radiation-induced pneumonitis, and ICI-induced myocarditis/cardiac failure. Further diagnostic evaluation should include infectious evaluation (urine, respiratory culture, viral culture/swab, blood cultures, serum galactomannan), CT imaging and consideration for bronchoscopy with bronchoalveolar lavage (BAL) +/– lung biopsy. This patient underwent CT imaging with findings as illustrated in Figure 2F. Infectious evaluation was negative for the presence of an infectious organism. Bronchoscopy with BAL was notable for the presence of chronic lymphocytes and macrophages with type-II pneumocyte hyperplasia. High-resolution CT chest is the imaging modality of choice, with common manifestations including ground-glass opacities or patchy nodular infiltrates, predominantly in the lower lobes (60). This should ideally be done with contrast, to rule out the presence of a pulmonary embolus. Five distinct types of radiologic abnormalities of CIP have been described, including cryptogenic organizing pneumonia (COP) like ground glass opacities, interstitial, hypersensitivity, and pneumonitis not-otherwise-specified (61).

#### Management

Corticosteroid therapy is the mainstay of CIP management, with >80% of CIP patients having their pneumonitis improve or resolve with corticosteroids alone. Pulmonology assessment is often warranted in any patient with suspected CIP, to evaluate for bronchoscopy or help to rule out alternative etiologies. In this patient with grade 2 CIP, ICI was temporarily held while the patient was treated with prednisone 1 mg/kg/d, followed by 5 mg/week taper over 4 weeks. Interval CT imaging at 4 weeks was notable for improvement in radiographic findings. In this case, since CIP resolved, the patient's ICI-therapy was restarted. While grade 1 or 2 CIP can be managed with low-dose corticosteroids and close observation, higher-grade CIP should be treated with high-dose corticosteroids (methylprednisolone IV 1–2 mg/kg/d). Infectious disease should be consulted in addition to the pulmonary team to rule out common or unusual infections, and in some cases, empiric antimicrobials may be given when infection cannot be completely excluded. Lung biopsies are typically not warranted, but may be useful rule out infection or lymphangitic tumor spread. Patients that do not demonstrate clinical improvement in CIP within 48–72 h should be considered for second-line therapies, options include infliximab, mycophenolate mofetil, IVIG, or cyclophosphamide. Retrospective studies have noted that up to 86% of CIP improves with corticosteroid treatment, however, of concern there was very poor response to additional immunosuppression (8, 62). In addition, a recent retrospective analysis has found that pneumonitis is associated with worse survival (63). There is currently a deficit in evidence for salvage treatment in steroid refractory patients. This is an area under current development to ameliorate the morbidity and mortality associated with respiratory-induced adverse events.

## Endocrine

### Case 1: Hypophysitis

*Clinical presentation: A 70-year old male with advanced renal cell carcinoma receiving Ipilimumab/Nivolumab, presents with new-onset fatigue and dizziness after 2 cycles of therapy*.

Immune-related endocrine events pose a clinical challenge as symptoms are often subtle. Patients can present with non-specific symptoms including nausea, vomiting, dizziness, headache, fatigue, and malaise. The pituitary, thyroid, pancreas, and adrenal glands are the organs most commonly affected, although parathyroid involvement has also been reported (64). The incidence of immune-related endocrinopathies was approximately 10% in a recent meta-analysis of 7,551 patients that received ICI (63). The risk of endocrine irAE is greatest with combination therapy, with rates of hypothyroidism (17%), hypophysitis (13%), and hyperthyroidism (10%) reported (4, 65).

#### Diagnostic Evaluation

In this particular patient, a physical examination was notable for intact visual fields, however, laboratory assessment showed mild hyponatremia, with both low TSH and free T4. Morning ACTH and cortisol were also low. MRI brain was notable for pituitary enhancement.

In patients with suspected hypophysitis, the pituitary-hypothalamic axis should be examined including free T4, TSH, LH, FSH, ACTH, and cortisol, as well as serum electrolytes. It is imperative to discern between primary vs. secondary hormonal deficiencies, as this will guide appropriate management. Clinicians should recognize that hypophysitis can result in secondary adrenal insufficiency and hypothyroidism. Failure to recognize this disease entity can have negative consequences for patient care; replacing thyroid hormone prior to cortisol repletion can precipitate adrenal crisis.

#### Management

Ipilimumab/Nivolumab therapy was temporarily held. In consultation with endocrinology, the patient was started on hydrocortisone 10 /5 mg in morning and afternoon, respectively. One week later he was started on a weight-based dose of levothyroxine. The patient was provided with “sick day” instructions for stress dosing of hydrocortisone and a medical alert bracelet. The patient demonstrated clinical improvement and was restarted on PD-1 monotherapy.

It is recommended that ICI is held for any-grade hypophysitis. Once patients demonstrate stability on hormonal replacement, ICI can be restarted. Higher-grade hypophysitis (grade 3+) can be managed with an initial pulsed dose of corticosteroids. Free T4 should be monitored for levothyroxine replacement. Key concepts of management include high index of clinical suspicion, appropriate localization of endocrine dysfunction, replacement of hormones and close monitoring. Immune-related endocrine events are unique as the manifestations are often irreversible and patients often require lifelong hormone replacement (66).

### Case 2: Hypothyroidism

*Clinical presentation: A 60 year old female patient with stage-III lung adenocarcinoma treated with durvalumab, has a thyroid stimulating hormone (TSH) of 8.5 mIU/l with normal free thyroxine (fT4). She was asymptomatic*.

Hypothyroidism is one of the most common irAEs from anti-PD-1, anti-PD-L1, and anti-CTLA-4 ICIs. A systematic review and meta-analysis by Barroso-Sousa et al. demonstrated that the overall incidence of hypothyroidism was 6.6% (65). Hypothyroidism can present with fatigue, unintentional weight gain, cold intolerance, constipation, myalgia, and dry skin.

#### Diagnostic Evaluation

Physical examination may be notable for goiter, bradycardia, diastolic hypertension, or delayed deep tendon reflexes. TSH and fT4 should be completed prior to initiation of ICI therapy and should be monitored every 4–6 weeks. It is important to differentiate primary from secondary hypothyroidism as discussed above, as well as differentiate hypothyroidism from late-phase thyroiditis. Elevated TSH with low fT4 is indicative of biochemical hypothyroidism. Upon detection, thyroid peroxidase (TPO) antibody should also be sent.

#### Management

Durvalumab therapy was continued. At 4 week follow-up, TSH level was noted to be elevated to 12 mIU/ml with normal fT4. She remained asymptomatic. However, given TSH >10 mIU/l, she was commenced on 75 mcg of levothyroxine daily. In patients with grade 1 hypothyroidism, ICIs may be continued with close monitoring of TSH and fT4. For grade 2 toxicity, appropriate thyroid supplementation should be administered with either continued ICIs or temporary withholding until symptomatic patients with any level of TSH elevation or in asymptomatic patients with TSH levels that persist >10 mIU/l (measured 4 weeks apart) improve. Grade 3 and 4 toxicities should be treated as grade 2 unless signs of myxedema (decreased mental status, hypotension, hypoglycemia, bradycardia, hypothermia) are present, in which case hospitalization for supportive therapy may be recommended. In general, TSH should be monitored every 6–8 weeks while titrating hormone replacement until a normal TSH is reached, with repeat testing annually or as clinically indicated.

## Hematology

### Case 1: Thrombocytopenia

*Clinical presentation: A 50-year old female with PD-L1*+ *metastatic lung adenocarcinoma presents with petechiae after 3 cycles of pembrolizumab treatment*.

Hematologic irAE that may occur from anti-PD-1/PD-L1/CTLA-4 include autoimmune hemolytic anemia, acquired thrombotic thrombocytopenia, hemolytic uremic syndrome, immune mediated thrombocytopenia, lymphopenia, and acquired hemophilia. Thrombocytopenia due to ICI is relatively infrequent, with reports ranging from 1 to 28% of patients (67–69).

#### Diagnostic Evaluation

In patients who develop thrombocytopenia during ICI therapy, other etiologies for this presentation should be considered, including bone marrow suppression, infiltration, platelet destruction, or platelet sequestration, with a differential diagnosis of myelodysplastic syndrome, disseminated intravascular coagulation, ICI-mediated thrombocytopenia, acquired thrombotic thrombocytopenia (TTP), and hemolytic uremic syndrome (HUS). A thorough history is important to evaluate for drug/toxin exposures or viral infections that may have led to thrombocytopenia. In this patient, CBC was notable for normal hemoglobin with grade 2 thrombocytopenia (platelets 70,000/μl). Renal function was normal. There was no evidence of platelet consumption or hemolysis on a peripheral blood smear. Hemolysis labs including serum lactate dehydrogenase (LDH), haptoglobin, indirect bilirubin, and CBC were normal. HIV, hepatitis B/C virus and *H. pylori* were negative. Thus, ICI-mediated immune thrombocytopenia was the most likely diagnosis.

#### Management

In this patient, ICI was held for 2 weeks, and a repeat CBC did not show improvement in platelet count until prednisone 1 mg/kg/dose was started. Re-evaluation at 2 weeks revealed improvement to grade 1 thrombocytopenia (Platelets 90,000/μl). Prednisone was tapered over 4 weeks, and the patient was able to be recommenced on pembrolizumab.

Most patients with low-grade thrombocytopenia improve with ICI withholding and initiation of oral corticosteroids. For higher-grade toxicities, a hematology service should be consulted for consideration of additional therapies for severe toxicity, such as IVIG, rituximab, cyclosporine A, mycophenolate mofetil, cyclophosphamide, or thrombopoietin receptor agonists. If indicated, IVIG initial dosing is recommended at 1 g/kg as a one-time dose which can be repeated if necessary (70).

## Gastrointestinal

### Case 1: Colitis

*Clinical presentation: A 45-year old male with advanced melanoma presents to the ED with abdominal pain, non-bloody diarrhea (*>*7 bowel movements per day) and fever 2 days after receiving 3rd dose of combination Ipilimumab/Nivolumab therapy*.

The most common GI toxicities reported from anti-CTLA-4 ICIs are diarrhea and colitis. Colitis is defined as inflammation of the lining of the colon, as opposed to diarrhea that is defined as increased number of bowel movements.

The incidence of colitis ranges from 8 to 27% with rates of diarrhea reported up to 54% of CTLA-4 treated patients. The incidence is greatest in patients treated with CTLA-4 monotherapy vs. CTLA-4/PD-1 combination therapy (71, 72). When PD-1 inhibitors were compared directly with CTLA-4 inhibitors, the relative risk of all-grade diarrhea and colitis was 0.58 and 0.16, respectively (73). Combination PD-1/CTLA-4 related deaths were recently found to be most frequently from colitis, accounting for 37% of fatalities (14). The time of onset is typically 5–10 weeks following initiation of therapy (74). ICI-induced hepatotoxicity has also been well-described, with incidence of 2–10% of patients receiving monotherapy (75) and 25–30% of patients being treated with combination PD-1/CTLA-4 therapy (75). Hepatitis has been found to be the second most common irAE leading to fatal outcomes anti–PD-1/PD-L1 therapy (14). The time of onset of hepatitis may also occur early in a patient's treatment course; typically commencing within the first 6–12 weeks after treatment initiation (76). Other less frequently reported GI toxicities include dysphagia, gastritis, duodenitis, and pancreatitis (77).

#### Diagnostic Evaluation

Diagnostic work-up for colitis includes standard laboratory testing to assess for infectious vs. non-infectious etiologies including CBC, CMP, ESR, and CRP. Stool cultures (bacterial, viral, ova, and parasites) should be obtained as well as stool calprotectin or lactoferrin to monitor disease activity, extrapolated from the management of inflammatory bowel disease. In patients with severe colitis, tuberculosis testing should be completed in potential preparation for the use of anti-TNF-α inhibitors for steroid-refractory colitis. Patients should also undergo radiologic imaging with a CT abdomen, which may show mesenteric vessel engorgement, colonic wall thickening, colonic distension, and pericolonic fat stranding (76). In severe cases, in those in which the diagnosis is uncertain, and to evaluate the severity of colitis, direct visualization of the colon with either a colonoscopy or flexible sigmoidoscopy should be considered. The presence of ulceration on direct visualization is associated with a corticosteroid-refractory course, and early infliximab can be considered in these cases. ICI-induced colitis has been reported to have greater predilection for descending colon (see Figure 2G) (71, 76). Endoscopy is also useful in monitoring colitis, particularly when resumption of therapy is being planned.

This patient underwent extensive evaluation as delineated above. CT imaging revealed thickening of descending colon with pericolonic fat stranding. Gastroenterology were consulted who completed colonoscopy, which demonstrated diffuse ulceration at the descending colon.

#### Management

The patient was managed with intravenous fluids and prednisone 1 mg/kg/day. This resulted in clinical improvement, with reduction in bowel movement to three times per day. The patient was continued on the initial corticosteroid dose for a further 4 days, and then steroids were tapered over 4 weeks. Upon completion of the prednisone taper, nivolumab monotherapy was resumed.

Patients with grade 2+ colitis should have ICI withheld, and treatment should only be resumed if toxicity improves to <grade 1. Cases of grade 3+ colitis often imply permanent discontinuation of anti-CTLA-4 therapy (74). Early intervention with corticosteroids and best supportive care with hydration, electrolyte repletion and antidiarrheal agents is crucial. If patients do not demonstrate adequate clinical response within 48–72 h of corticosteroid therapy, TNF-blocker therapy with infliximab should be commenced. This can be repeated again at a 2-week interval. Vedolizumab, an anti-integrin α4β7 antibody, should be considered in patients who develop colitis that is refractory to infliximab or in cases that anti-TNF- α therapy is contraindicated.

## Discussion

Currently, there is a paucity of literature on the precise immunopathogenesis of specific irAEs, however several mechanisms have been suggested that vary depending on the irAE in question.

A mechanism for irAE development due to enhanced T cell activity against shared antigens across normal and cancer cells is supported by several preclinical models in autoimmune myocarditis and autoimmune dilated cardiomyopathy (78, 79). In addition, abrogation of PD-1 pathway may contribute to the pathogenesis of rheumatoid arthritis and giant cell arteritis (80, 81). Furthermore, vitiligo is the most common cutaneous irAE in patients with melanoma, likely as a consequence of shared antigens on melanoma cells and melanocytes (82). Elevated levels of inflammatory cytokines are also likely involved in the pathophysiology of irAE. Both PD-1 and CTLA-4 therapies have been found to promote Th1- and Th17-mediated immune responses resulting in elevated circulating levels of IL-17 and IFN-γ (83–85). Insights into the pathogenesis of ipilimumab-induced enterocolitis has facilitated the use of steroid-sparing agents such as infliximab and vedolizumab in this particular irAE (86, 87). Another potential mechanism is that of increased levels of pre-existing autoantibodies seen with ICI-induced myasthenia gravis, autoimmune hemolytic anemia, autoimmune thyroiditis and type 1 diabetes mellitus (88–92). Finally, CTLA-4 therapy has been found to cause hypophysitis, likely due to enhanced complement-mediated inflammation due to direct binding of anti-CTLA-4 antibody with CTLA-4 expressed on normal pituitary tissue (93). Further insights would allow the development of personalized and targeted therapies for patients, potentially reducing exposure to prolonged immunosuppression, thereby enhancing efficacy and reducing toxicity of treatment.

The association of irAE with efficacy of ICI therapy is also under investigation. As detailed previously, development of vitiligo has demonstrated a significant association with beneficial clinical outcomes (39). A recent multicenter retrospective study of patients with advanced NSCLC treated with nivolumab monotherapy found that the development of irAEs was a strong predictor of survival outcomes (94). Indeed, patients who experienced two or more irAEs had a more pronounced survival benefit, suggesting a mechanistic association between irAEs and immunotherapy efficacy. These findings have not been universally confirmed, but certainly raises the question of whether the degree of immune activation is associated with superior treatment effect.

Risk factors for the development of irAEs are also being evaluated. The clinical manifestations, timing, and severity of irAE appear to depend on immunotherapy regimen, with increased frequency and severity demonstrated with combination therapies (4, 5, 65). Patients with pre-exiting autoimmune disease appear to be at increased risk of irAE. A number of retrospective studies have indicated that while patients with pre-existing may be at increased risk for both exacerbation of their autoimmune condition and for *de novo* immune-related adverse, this should not preclude treatment (95). Studies regarding other risk factors for developing irAE are lacking. There is likely a genetic predisposition to irAE given the role of genetics in autoimmune disease, indeed, in PD-1 deficient mice, genetic backgrounds were found to have different degree and divergent autoimmune conditions (79, 96, 97). Cappelli et al. recently evaluated immunogenetics in patients with ICI-induced IA and found that patients of European descent were more likely to be positive for HLA-DRB1 shared epitope alleles than population controls (98). Furthermore, patients with CTLA-4 gene variant 1661A>G may predispose melanoma patients to the development of endocrine irAEs (99). In addition, the JHH group have recently demonstrated that in NSCLC, tumor histologic type may be a risk factor for CIP development and that the development of CIP worsens survival in patients receiving immunotherapy (58, 59).

A new wave of research is focused on identification of irAE biomarkers, which would enable identification of populations at higher risk for development of irAE. Both obesity and smoking result in pro-inflammatory state (100–102). An “inflamed phenotype” may exist that could affect both therapeutic responses and toxicity risk. It may also be of benefit to account for the baseline inflammatory profiles present in the patient prior to ICI, but this is an area that requires additional research. The risk of both therapeutic and adverse effects of ICI may also be modulated by the gut microbiota (103). Preclinical studies have found that *Bifidobacteria* promote the natural antitumor immune responses, possibly by inducing a favorable shift toward Th1 responses (104). Patients treated with CTLA-4 therapy with a predominance of bacteria from the *Bacteroidetes phylum* were found to have reduced rates of ipilimumab-induced colitis (105). These findings raise the possibility that variations in gastrointestinal flora that affect host immunity influence the risk of irAE. Indeed, differences in the intestinal microflora of patients may explain some of the heterogeneity in immunotherapeutic and toxicity outcomes in patients receiving ICI therapy. A number of therapeutic avenues are currently being explored. Anderson and Rapport recently suggested a biomarker profile incorporating IL-17/IFN-γ/IL-10/TGF-β1/CRP based on findings from ipilimumab-associated colitis (106). Other potential biomarkers include measurement of circulating neutrophil activation using myeloperoxidase/matrix metalloproteinase 9/L-selectin (107).

## Conclusion

Immunotherapy is associated with a unique side effect profile that can result in significant morbidity and mortality. Immune-related toxicities require prompt recognition and intervention to optimize clinical outcomes. As immunotherapy enters common clinical practice, it is crucial that multidisciplinary collaborations are established to improve the recognition and management of common or serious irAE, in particular. In this emerging field, an anti-cancer therapy that is able to selectively kill cancer cells without causing toxicities to normal cells, remains elusive. Further research is required to enhance our understanding and to define the immunopathogenesis of irAE so that strategies for prevention, early detection and targeted therapy can be developed.

## Consent

Written and informed institutional consent was provided for the use of clinical images. The clinical cases presented in this review are fictional.

## Author Contributions

This review was drafted by CC and KB and critically revised by JN.

### Conflict of Interest Statement

The authors declare that the research was conducted in the absence of any commercial or financial relationships that could be construed as a potential conflict of interest.
